# Assessing paver screed compaction units and their impact on asphalt quality parameters through field trials

**DOI:** 10.1038/s41598-026-57397-2

**Published:** 2026-06-10

**Authors:** Leandro Harries, Stefan Böhm, Jia Liu

**Affiliations:** Institute of Transportation Infrastructure Engineering, Technical University of Darmstadt, Darmstadt, Germany

**Keywords:** Asphalt, Paving, Compaction, Paver, Tamper, Vibratory unit, Quality, Engineering, Materials science

## Abstract

Ensuring the durability and longevity of asphalt pavements under traffic loads requires effective compaction during paving to resist deformation and withstand environmental stresses. While previous field-based studies have addressed asphalt compaction and selected screed settings, this study specifically links tamper speed and vibratory unit speed to pre-compaction, vertical void distribution, aggregate structure, and 3D surface roughness under real paving conditions. Two field trials using an ABG P6820D paver and BOMAG BW 174 roller were conducted with an AC 16 BN asphalt mix. Laboratory analyses evaluated layer thickness, bulk density, cavity structure, and surface roughness. The findings highlight that tamper speed significantly influences layer thickness and degree of compaction, while vibratory unit speed showed no substantial effect. Vertical void distribution was highly homogeneous (> 98%) across pre-compacted areas, indicating tamper speed does not affect void distribution. Surface roughness tended to decrease with increased tamper speed, but vibratory unit speed showed no measurable benefit. No significant correlation was found between grain-to-grain distance or microstructural properties and vibratory unit speed. The study recommends minimizing adjustments to tamper speed during paving and suggests switching off the vibratory unit for the investigated AC 16 BN mixture under comparable paving conditions, provided that the required pre-compaction and surface quality are achieved. Regular monitoring of tamper wear and precise pre-paving adjustments are essential to enhance pavement quality and reduce maintenance costs.

## Introduction

To ensure the durability of asphalt pavements under traffic loads and prevent harmful deformation, it is crucial to provide adequate resistance to deformation and facilitate effective load transfer to the underlying layers. The pavement’s ability to resist deformation and withstand stresses from temperature and weather conditions largely depends on the quality of compaction. Poor paving practices and inadequate compaction significantly impact the lifespan and strength of asphalt pavements, leading to issues such as premature cracking, rutting, or material breakdown^1,2^. Errors in setting up the asphalt paver can result in defects such as aggregate crushing due to excessive compaction, bitumen accumulation caused by overly strong vibration, or waves in the paved layer caused by a negative screed angle of attack. Such defects can reduce the pavement’s life cycle and lead to unnecessary costs and environmental consequences^3^.

Achieving proper compaction generally involves two key stages: initial compaction by the screed of the paver, followed by final compaction using rollers. The initial compaction stage is critical, as defects introduced at this stage are difficult to correct through subsequent roller operation. Paving hot mix asphalt (HMA) is a complex process that requires the compaction units of the screed, including tampers and vibratory units, to be adjusted to the prevailing paving conditions. In addition to the asphalt mix design and the target layer thickness, external and variable factors such as mix temperature, ambient temperature, wind conditions, paving speed, delivery consistency, and support from the underlying layer must be considered when setting up the paver. If these factors are not properly accounted for, the resulting mix performance may be affected through insufficient or excessive initial compaction, inhomogeneous air void distribution, aggregate crushing, bitumen accumulation, surface irregularities, reduced durability, and a shorter pavement life cycle.

Despite these efforts, optimal paving results are often difficult to achieve due to the high dependence on the experience of paving personnel. As noted by Utterodt^4^, even decades after fundamental research into the effects of tamping and vibration on asphalt layers, there remains a lack of scientifically validated guidelines for the ideal combinations of parameters like tamper frequency, vibration frequency, and paving speed. This lack of validated recommendations also extends to the settings of high-compaction screeds.

It is therefore crucial to investigate the influence of various screed settings on paving quality, as this plays a significant role in the practical application of asphalt. To achieve this, field trials were conducted to evaluate screed compaction settings under real paving conditions. While previous field-based studies have addressed asphalt compaction and selected screed settings, the present study focuses on linking tamper speed and vibratory unit speed to layer thickness, degree of pre-compaction, vertical void distribution, aggregate structure, and 3D surface roughness. This provides a more detailed assessment of how individual screed compaction units affect asphalt quality parameters in practice.These field trials are crucial for validating theoretical findings under real-world conditions and ensuring their practical applicability. With the increasing automation of the paving process, the reliance on operator experience will diminish, necessitating scientifically validated insights. Research in this area is essential to optimizing pavement longevity, minimizing maintenance costs, and ultimately alleviating financial burdens on public budgets.

## Literature study

**Fig. 1:**
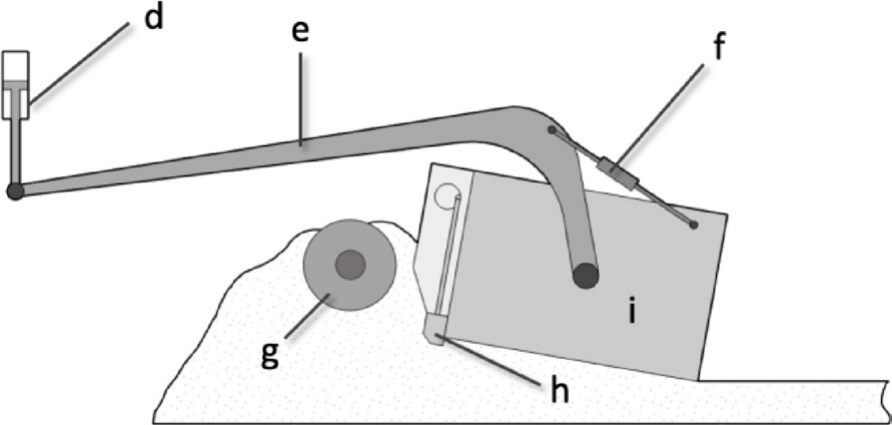
Cross section of paver screed (**d**): tow point rams; (**e**): tow arm; (**f**): lifting cylinder; (**g**): auger; (**h**): tamper; (**i**): screed).

For paving projects of various sizes, from small parking lots to multi-lane highways, asphalt pavers are widely used for road construction. The primary tasks of an asphalt paver involve collecting the HMA from the material hopper, spreading it evenly across the paving width using an auger that operates perpendicular to the travel direction (see legend ‘g’ of Fig. 1), and then smoothing and pre-compacting it with the screed (see legend ‘i’ of Fig. 1) to achieve uniform paving with the desired layer thickness and density. During operation, the screed is pulled by two tow points and rests freely on the asphalt, a setup known as a “floating” screed. Pre-compaction is achieved by the weight of the screed itself. In many cases, depending on the manufacturer and region (outside of North America), additional compaction units are integrated. Standard screeds include a tamper (see legend ‘h’ of Fig. 1) and a vibration unit within the screed assembly. Guideline-based recommendations for screed settings are often specified according to the asphalt mixture type. As an example, the Vögele Paving Guide^11^ recommends a tamper speed of 600 to 1000 rpm and a vibratory unit speed of 1800 to 2100 rpm for comparable AC 16 mixtures.

In asphalt construction with pavers, numerous studies, both past and recent, have employed various methods, including laboratory tests (e.g^5^, mathematical modeling (e.g^6^, and simulations (e.g^7^, to examine this subject. These approaches offer insights into various mechanisms but are limited in scope, as only the actual paving process can account for all real-world influences and enables investigations on actual drill cores. In the literature publicly available studies utilizing large-scale field tests to analyze the mechanisms of compaction units remain scarce.

###  Tamper unit

During operation, the tamper performs an oscillating vertical movement $\:y$ according to the general Eq. (1), where $\:A$ is the amplitude, $\:f$ is the frequency and $\:t$ is the time. This leads to the mix being compressed as the tamper moves downwards.

1 $$\:y\left(t\right)\:=\:A\cdot\:\mathrm{s}\mathrm{i}\mathrm{n}\left(2\pi\:ft\right)$$

In addition to the tamper frequency, the tamper stroke can also be adjusted on some screeds. However, adjusting the stroke can be time-consuming. Some manufacturers offer automatic adjustment of the stroke.

Böhmer^5^ used a scaled-down screed simulator in the lab to study the compaction of a AC 30, finding that the degree of compaction increases with tamper frequency, especially at lower tamper strokes, where the relationship is nearly linear. However, grain fragmentation occurs at higher strokes and frequencies, and saturation may be reached with further increases. In field trials^8^, a linear relationship between tamper frequency and compaction was observed, with a Coefficient of Determination (CoD) of 0.9836, although raising the frequency from 360 to 1440 rpm resulted in just a 1.2% increase in compaction. Jia et al.^9^, using a dynamic model of vibratory compaction, found that increasing paver speed decreases compaction due to fewer tamper strokes per unit length. They also showed that compaction peaks at 5 Hz and 17 Hz tamper frequencies, which contrasts with findings from^5] and [8^, where higher frequencies continued to enhance compaction.

### Vibratory unit

The vibration unit works on the principle of surface vibration. Unbalanced masses are stimulated to vibrate to generate mechanical vibration^10^. Depending on the manufacturer, there are several unbalanced mass bars in the area of the base screed and the extension parts.

According to common opinion, such as in the Vögele Paving Guide^11^, the vibration unit is said to have little to no influence on the compaction performance of the paver screed. Böhmer^5^ was also able to prove a correlation between the vibration unit and compaction success using the aforementioned downscaled screed simulator. However, it should be noted that the tests were carried out with the tamper switched off. He was unable to determine a peak range depending on the change in exciter frequency or unbalance torque. An increase in the excitation frequency or the unbalanced mass always led to an increase in the degree of compaction k. Investigations from Kapustin^12^(cited in^5^ in the range from 50 Hz to 500 Hz on an asphalt concrete also showed no optimum frequency range. Böhmer^5^ points out that excessively high frequencies could lead to lifting of the screed and a damaging load on the unbalance bearings due to the excessive excitation force.

Wan and Jia^6^ studied how excitation frequency affects compaction efficiency by developing a Kelvin-Voigt spring/damper model to simulate the vertical and horizontal movement of the mix. Their simulations and field tests, using AC-25 (based on the Chinese standard JTG F40-2004), revealed a peak in density increase when the excitation frequency was raised. This partially contradicts Böhmer’s^5^ findings, which suggested a constant increase in density with rising frequency. Below the screed’s resonant frequency, density increased rapidly, but slowed down above it. They also noted that the vibration unit’s effect on compaction decreases with higher tamper frequencies. At 19 rad/s, a 4% compaction increase was observed, while at 57 rad/s, the increase was only 2%.

The findings from the literature therefore do not allow a clear conclusion to be drawn regarding the influence of the vibratory unit. While practical guidance^11^ assumes little to no influence on the compaction performance of the paver screed, Böhmer^5^ and Wan and Jia^6^ were able to show an influence under certain test conditions. However, the results are only comparable to a limited extent, as the studies differ in terms of asphalt mix, screed configuration, excitation range and, in particular, whether the tamper was switched on or off. It can therefore be assumed that the effect of the vibratory unit depends strongly on the interaction with the overall screed system and cannot be assessed solely on the basis of the vibration frequency.

## Trial paving

Investigating the influence of compaction units on asphalt in a laboratory setting presents significant challenges and can only be approximated using scaled-down paving machines, even with considerable effort. Consequently, conducting studies on full-scale test pavements is recommended, as these provide the most accurate representation of real-world construction scenarios. However, such studies are both material- and labor-intensive, underscoring the importance of well-planned paving setups during preliminary stages.

For the field trials, an ABG P6820D paver equipped with a VB 78 screed featuring a single tamper, along with a BOMAG BW 174 roller, was employed. The paver maintained a consistent standard paving speed of 3.5 m/min throughout the trials.

Information on the asphalt mixes used, can be found in Tables 1 and 2. The binder content was determined following the German regulation TP Asphalt-StB Part 1^13^, the maximum density following TP Asphalt-StB Part 5^14^, and the reference bulk density in accordance with TP Asphalt-StB Part 6^15^. The cumulative passing weights in Table 2 were determined in accordance with TP Asphalt-StB Part 2^16^. The information on the binder type, the proportion of reclaimed asphalt used and the aggregate type were taken from the initial test reports of the asphalt mixing plant.

**Table 1:** General asphalt mixture characteristics for the asphalt mixes AC 16 BN and AC 16 BN (R).

HMA Properties	AC 16 BN	AC 16 BN (*R*)
Binder Type	50/70	50/70
Binder Content	4.2% of mass	4.6% of mass
Reclaimed Asphalt	0% of mass	20% of mass (16 RA 0/1)
Aggregate Type	Diabas/Basalt	Quartzite
Aggregate Shape	Mainly Cubic Shaped	Mainly Cubic Shaped
Maximum Density	2.701 g/cm^3^	2.522 g/cm^3^
Reference Bulk Density	2.581 g/cm^3^	2.385 g/cm^3^

**Table 2:** Information on the grading curve for the asphalt mixes AC 16 BN and AC 16 BN (R).

Sieve Size [mm]	Cumulative Passing Weight [% of mass]	
	Trial Paving I	Trial Paving II
	AC 16 BN	AC 16 BN (*R*)
22.4	100.0	100.0
16.0	97.7	97.3
11.2	75.0	74.0
8.0	59.2	59
5.6	47.6	46.7
2.0	30.0	33.1
0.125	9.1	8.4
0.063	7.0	6.7

### Trial paving I

**Fig. 2:**
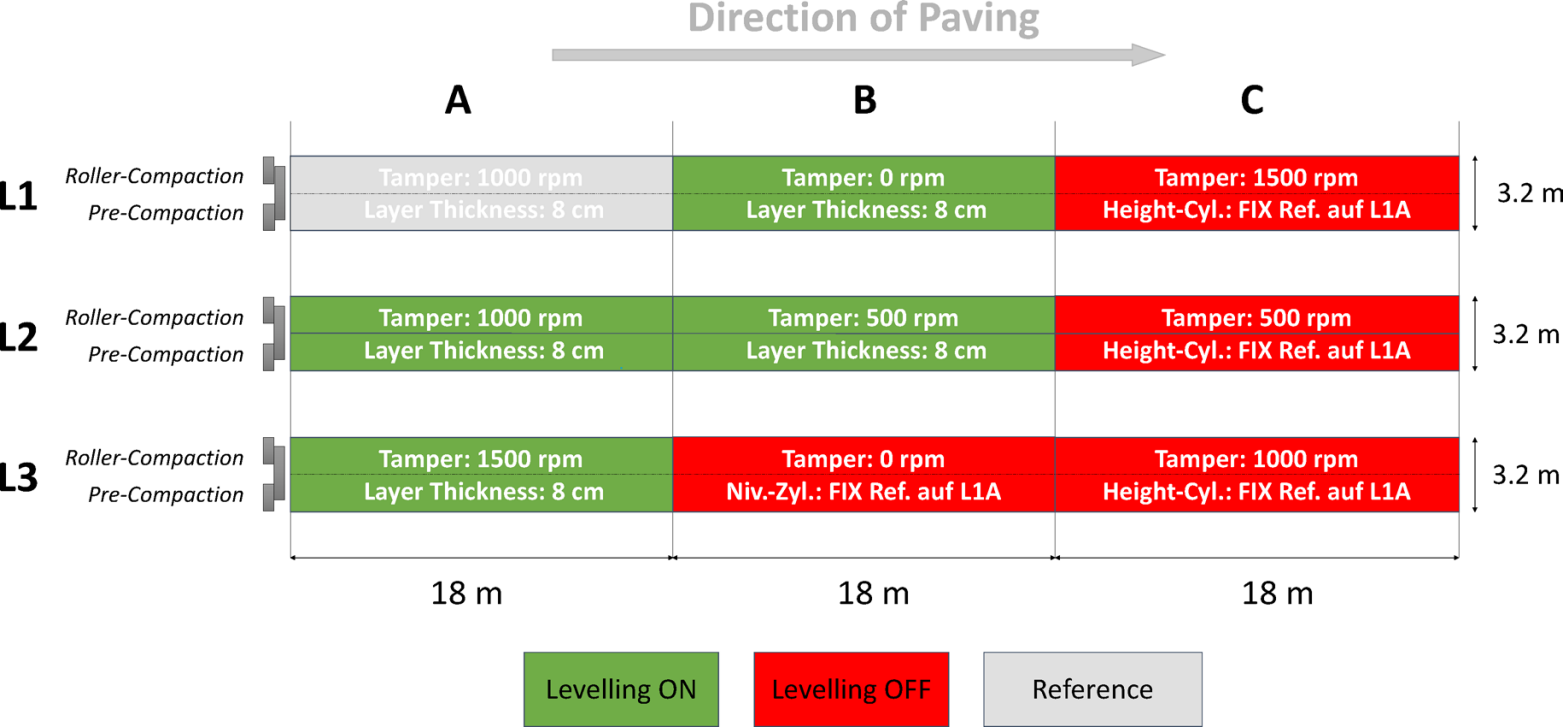
Trial paving spring 2023 - paving setup A.

The paving plan for the tamper tests is shown in Fig. 2. A total of three tracks, each with three sections, were paved. Each section has a length of 18 m. According to^11^, it can be assumed that a screed is leveled out after approx. 15 m. The green fields indicate the sections in which a levelling system was used and a layer thickness of 8 cm was targeted. In the red fields, the height of the tow points was kept constant so that a corresponding layer thickness is being reached depending on the setting of the tamper. The fixed value for the tow point was determined by a reference value in section A of lane L1. The paver came to a standstill on the last meter of the L1 and L2 paving lanes and the screed was immediately blocked. After a waiting time of around five minutes, the screed was slowly raised to its maximum height and the paver was carefully driven off.

The tamper frequency was systematically varied in four stages from 0 rpm to 1500 rpm. This covered the entire spectrum of possible tamper speeds of the VB 78 screed. The vibration unit was switched off during the entire paving process. It should be noted that the vertical position of the tamper in relation to the screed plate is unclear when the tamper is switched off and may not be uniform across the width of the screed.

### Trial paving II

**Fig. 3:**
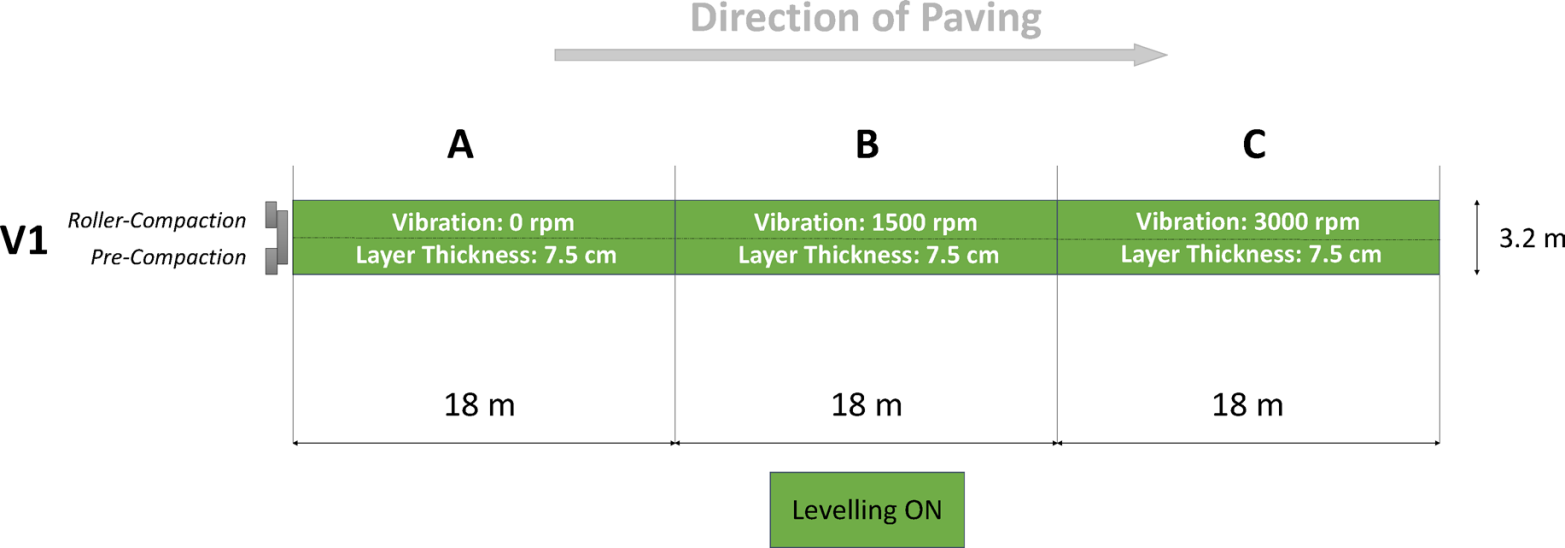
Trial paving autumn 2023 - paving setup B.

The setup of field trial II (cf. Figure 3) is much less complex. Due to the expected smaller differences in the vibration settings, only one lane with three sections was paved. The levelling system was set to a paving thickness of 7.5 cm instead of 8.0 cm in order to come as close as possible to the actual layer thicknesses of field trial I, which resulted after the evaluation (cf. Section  5.1).

The frequency of the vibration unit was systematically varied in three stages from 0 rpm to 3000 rpm. This covered the entire spectrum of possible vibration unit speeds for the VB 78 screed. The tamper speed was set to a constant 1000 rpm.

### Asphalt sample extraction

**Fig. 4:**
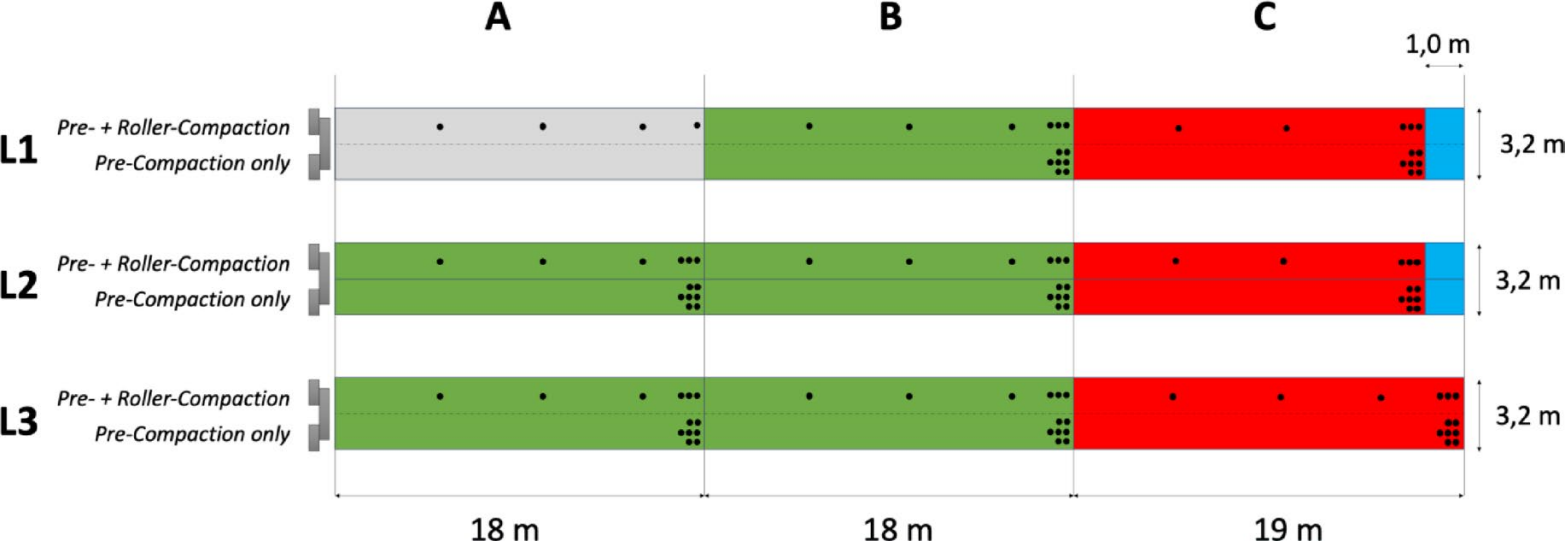
Asphalt sample extraction plan for field trial I in Bad Hersfeld, Germany.

**Fig. 5:**
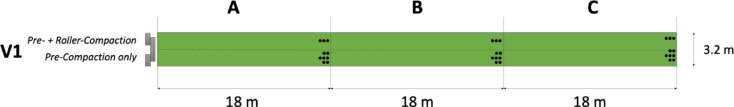
Asphalt sample extraction plan for field trial II in Limburg, Germany.

The sampling concepts are shown in Fig. 4 for field trial I and in Fig. 5 for field trial II. The black dots indicate the actual extraction point. These were positioned in such a way that the main quantity of cores was taken in the area where the base screed and the extending part of the paver screed were in contact with the HMA.

The analysis was carried out on the three cores taken in series on both the pre-compacted and the roller-compacted side. The blue areas at the end of paving lanes L1 and L2 (cf. Figure 4) show where the asphalt slabs were extracted.

**Fig. 6:**
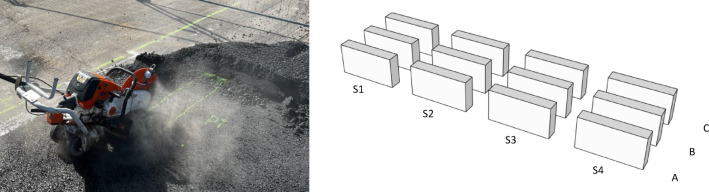
left - Asphalt slab extraction using a stone saw; right - Cutting pattern of the removed asphalt slab.

The asphalt slabs were extracted using a mobile stone saw (cf. Figure 6, left). To facilitate the extraction, the underlying asphalt layer was covered with sand beforehand to prevent it from sticking. Nevertheless, difficulties were encountered, so that only the removed slab of paving lane L2 could be used for further analysis. In the laboratory, the sample was cut into four sections, each with three further cuts, to achieve proper sizing for analysis (cf. Figure 6, right).

## Methodology

**Fig. 7:**
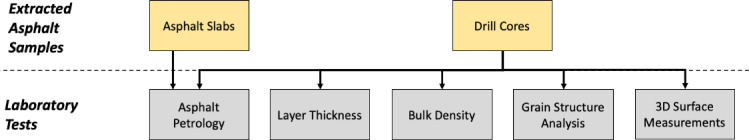
Overview of laboratory test program (asphalt specimen from field trials I and II).

The influence of the tamper speed and the influence of the vibratory unit speed on the paving results were investigated on the basis of laboratory tests on the asphalt samples taken. Initially, conventional parameters such as layer thickness and bulk density were used to analyze the influence of the compaction units followed by further evaluation methods (cf. Figure 7).

The Least-Significant-Difference (LSD) test according to^17^ was carried out for the statistical evaluation and to assess whether mean values of results differ statistically significant with an alpha of 5% from one another.

### Conventional parameters

The measurement of the layer thicknesses and the determination of the bulk densities of the drill cores taken from the field trials I and II was carried out in accordance with TP Asphalt-StB Part 8^18^.

### Asphalt petrology

**Fig. 8:**
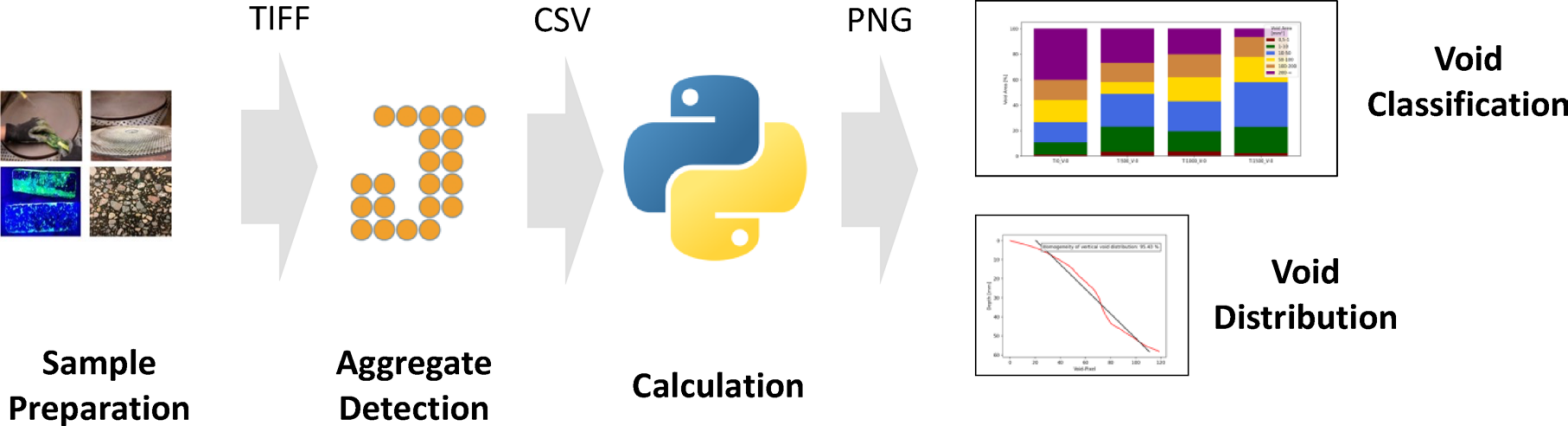
Procedure for analyzing the cavity structure using asphalt petrology methods.

Asphalt petrology applies methods from rock science to asphalt as a construction material^19^. The samples are prepared and scanned using the procedure described below. The high-resolution image is then analyzed using the JMicroVision^20^ software. Using color thresholding, the now fluorescent cavities can be differentiated from the rest of the asphalt. The individual cavity objects are generated by an object recognition algorithm based on contiguous pixels that are classified as cavities. The software calculates a variety of geometric data for the objects, which can be exported for further processing. The procedure is shown schematically in Fig. 8.

**Fig. 9:**
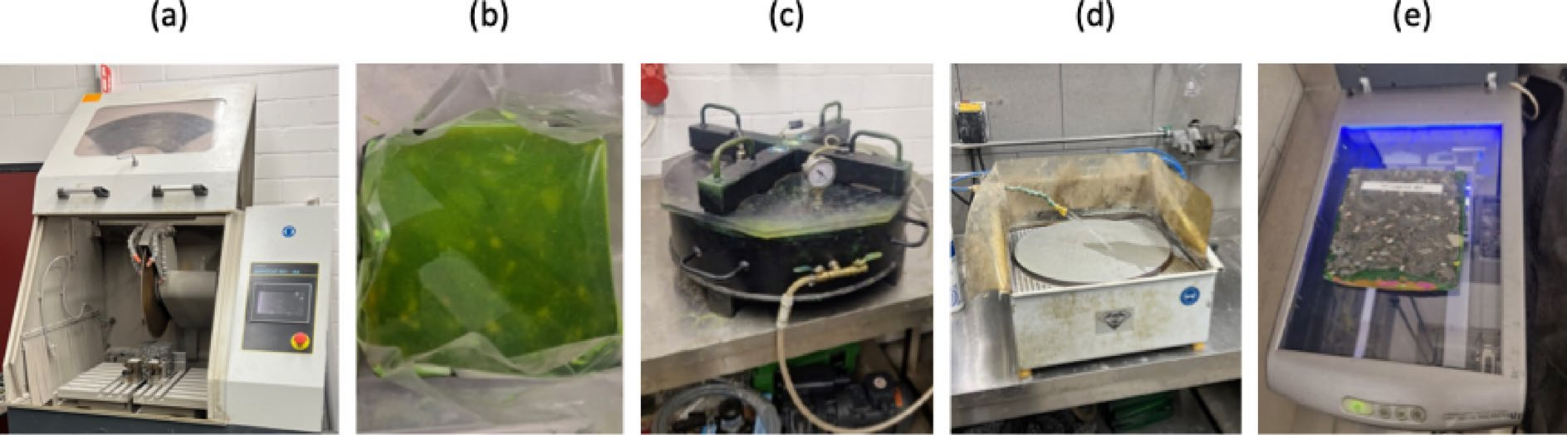
Procedure for conducting an asphalt petrological examination.

To carry out the asphalt petrological tests, the samples were first cut to size using an automatic precision saw (cf. Figure 9 (a)). The cut specimens were then placed in a mold made of foil, which lies as close as possible to the lateral and lower surfaces (cf. Figure 9 (b)). In the next step, the dried specimens were exposed to fluorescent epoxy resin in a vacuum chamber, which can penetrate into the smallest cavities due to the negative pressure (cf. Figure 9 (c)). After curing, the excess resin was grinded off so that epoxy resin remains only in the cavities and the surface is as even as possible (cf. Figure 9 (d)). The finished test specimens were scanned using a flatbed scanner that has been converted to UV LEDs (cf. Figure 9 (e))^21^.

Two algorithms for quantitative evaluation are used in these investigations. The first algorithm enables the proportion of defined cavity classes in mm^2^ to be calculated and displayed graphically. This allows the effect of different installation conditions on the cavity size distribution to be determined.

The second algorithm can be used to quantify the homogeneity of the vertical cavity distribution. To do this, all cavity pixels are first added up line by line so that a sum line is obtained. In the second step, a linear regression is carried out, which specifies the ideal line of the distribution, as a linear relationship between the depth of the test section and the summed void pixels would mean that there are the same number of void pixels in each line. Finally, the deviation of the actual line from the ideal line is calculated and the corresponding plot is plotted.

**Fig. 10:**
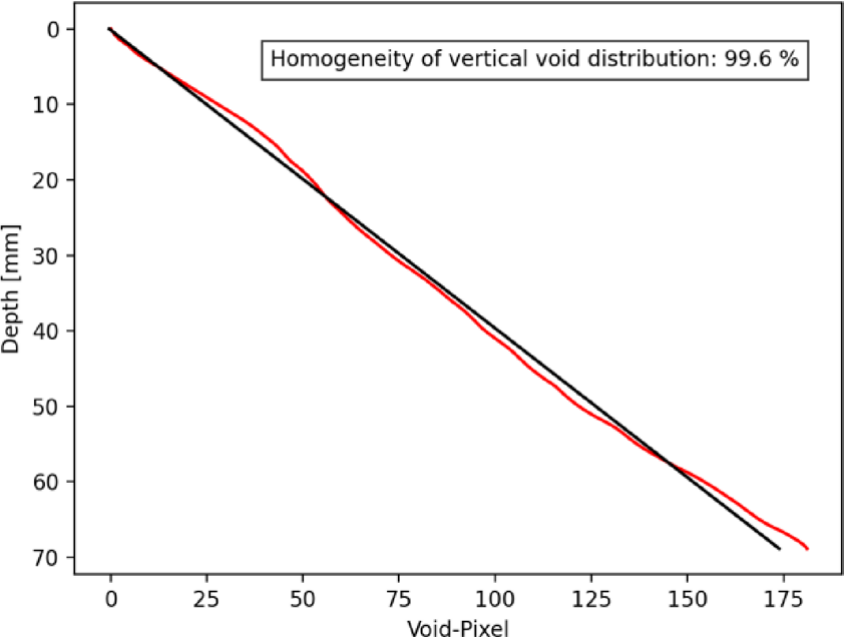
Example of the vertical cavity distribution for field trial I lane L2 Section B.

As an example, to illustrate the vertical void distribution a plot for the evaluation of lane L2 section B of field trial I (tamper speed 500 rpm; leveling switched on) is shown in Fig. 10. The sum line of the voids (red line) is almost linear and hence very close to the ideal line (black line). The vertical homogeneity of the cavity distribution is therefore close to 100%.

### 3D surface measurements

**Fig. 11:**
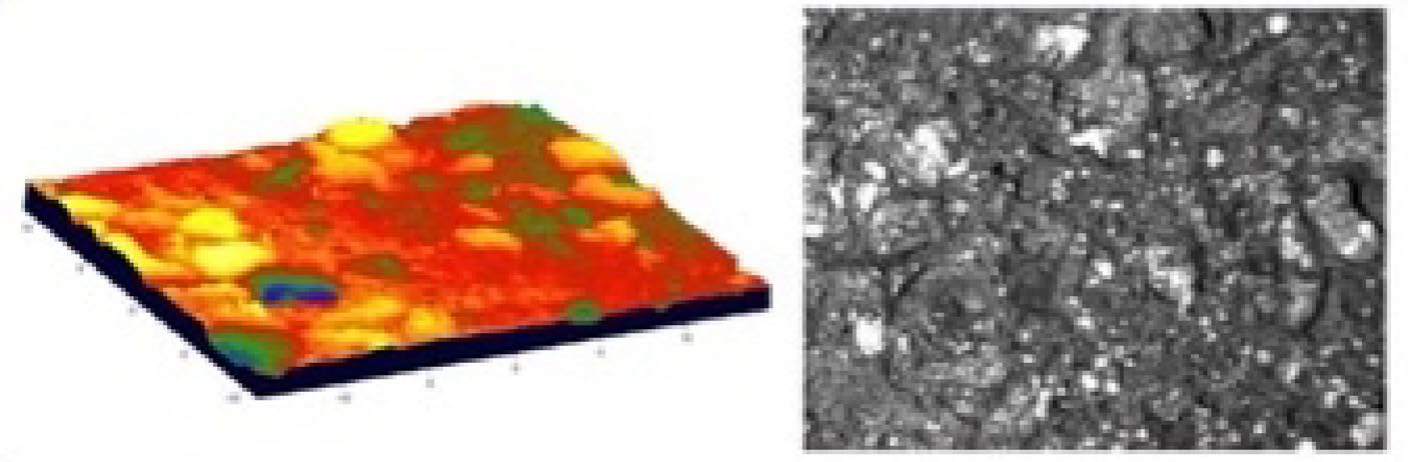
Example of 3D surface image - left: 3D image of surface; right: photo of asphalt surface.

The surface roughness of an asphalt surface can be determined using the fringe projection method. In this process, fringe patterns are projected onto a surface with a light source and captured by one or more cameras. The distortion of these stripes on the surface makes it possible to determine the shape and structure of the object. The captured images are then processed to create a detailed 3D model. Using a high-resolution camera detailed images of surfaces in the µm range are possible. An exemplary image of an asphalt surface is shown in Fig. 11 on the right. The corresponding elevation image can be seen in Fig. 11 on the left.

Based on the detailed images, elevation images of surfaces can be created and roughness values can be calculated using the LMI Technologies software^22^. The average roughness value S_a_ over the entire surface, the maximum roughness value S_max_ and an average roughness value from the five largest peaks and valleys S_z_ can be determined. The roughness values are output in µm by default.

**Fig. 12:**
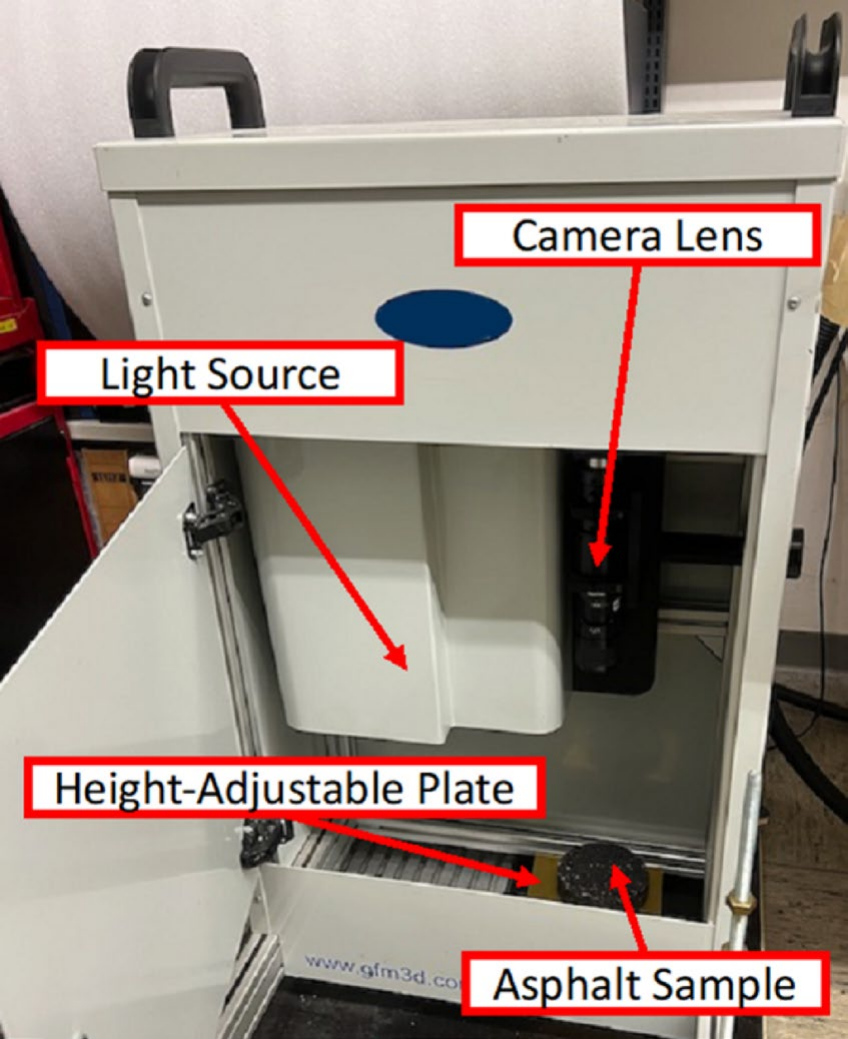
MikroCAD 3D surface measurement system.

The MikroCAD^22^ surface measurement system from GFM was used to carry out the surface roughness measurements (cf. Figure 12). To carry out the measurements, the surfaces of the drill cores were positioned at the correct distance from the camera. This was ensured via a live image within the measurement software. The height was set using a height-adjustable plate located inside the camera system.

## Results

### Layer thickness and degree of compaction

**Fig. 13:**
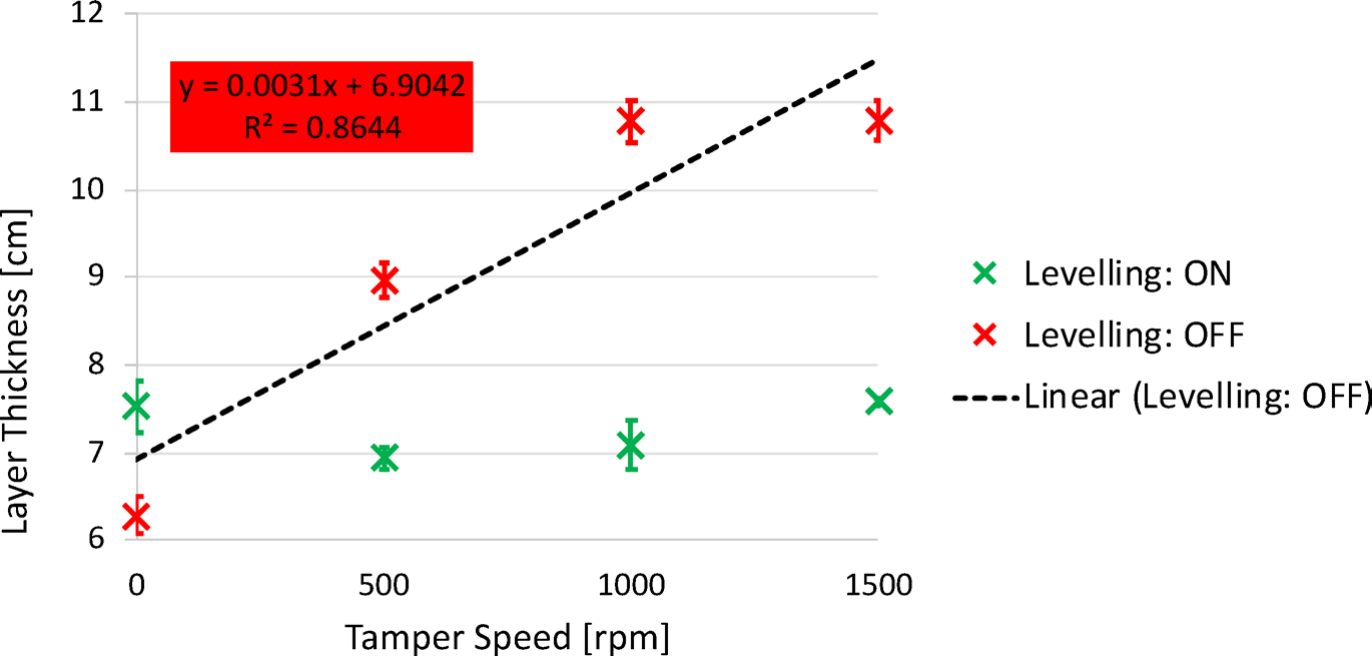
Field trial I – Layer thickness (pre-compacted) as a function of the tamper speed.

Figure 13 shows the results of the layer thickness measurement as a function of the tamper speed. With the leveling system switched off, there is a linear increase in layer thickness in the increase range from 0 rpm to 1000 rpm. This is consistent with the general understanding that increasing the tamper speed leads to more material getting under the screed^11^. When increasing from 1000 rpm to 1500 rpm, saturation seems to occur. This is probably due to the fact that the angle of attack decreases proportionally with the increase of layer thickness and therefore no or only a very small amount of additional material gets under the screed. With the leveling system switched on, a layer thickness of 8.00 cm should result. However, as can be seen in Fig. 13, this was not achieved in all sections. Unfortunately, the paving personnel were unable to achieve the targeted constant layer thickness of 8 cm. A major discrepancy results from the difference in the layer thickness at the tamper speed of 1000 rpm. As the reference field was also installed at a tamper speed of 1000 rpm, the layer thicknesses should be the same here. This is due to the fact that problems with the leveling system occurred in the reference section and the target layer thickness in the reference field of 8 cm was far exceeded at 10.6 cm. The results are therefore comprehensible.

**Fig. 14:**
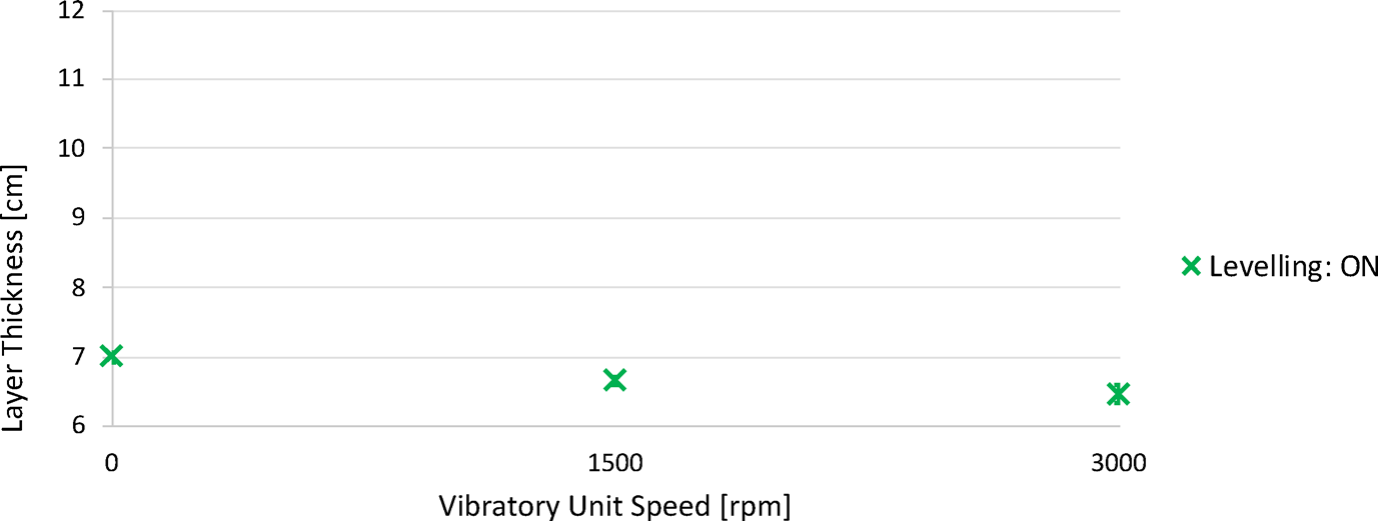
Field trial II – Layer thickness (pre-compacted) as a function of the vibratory unit speed.

As part of field trial II, a target layer thickness of 7.5 cm was aimed for the reasons mentioned in Sect.  3.2. Unfortunately, this was also consistently undershot and the layer thickness could not be kept constant by the paving personnel (cf. Figure 14).

**Fig. 15:**
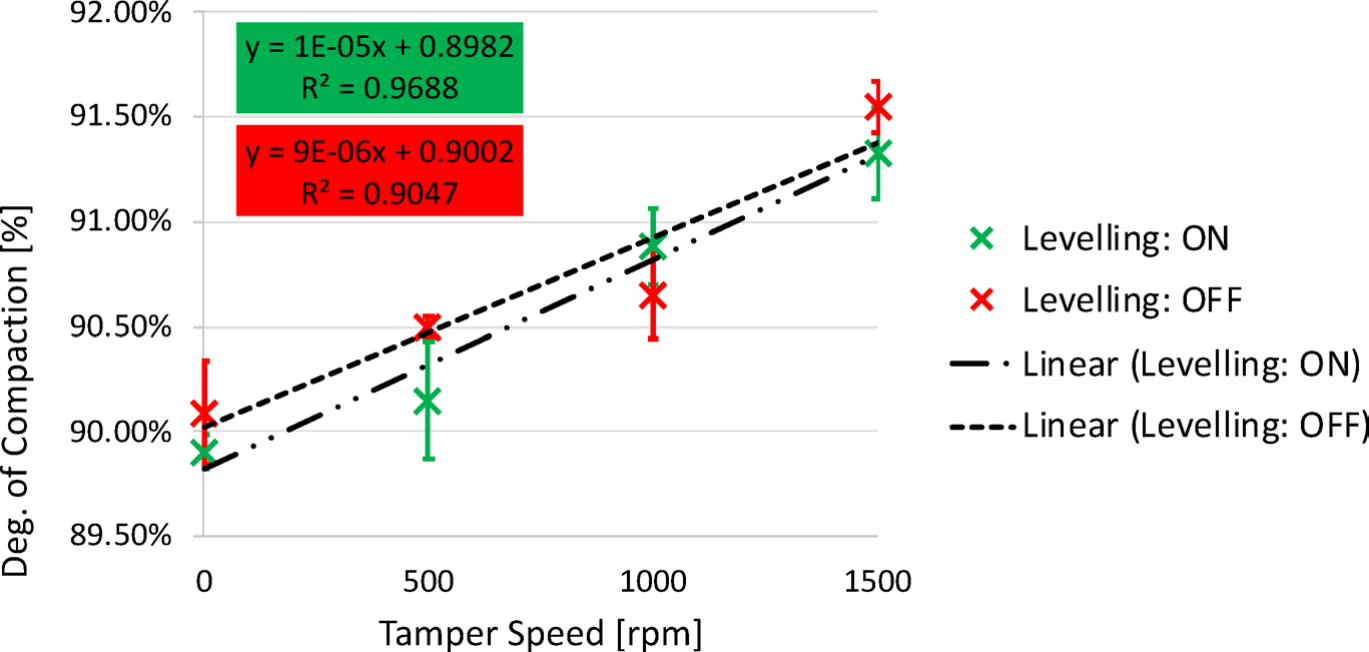
Field trial I – Degree of compaction (pre-compacted) as a function of the tamper speed.

The results of the degree of compaction as a function of the tamper speed can be found in Fig. 15. A generally increasing trend between tamper speed and the degree of compaction can be observed. This trend is visible both with the leveling system switched on and switched off, although not all differences between individual settings are statistically significant. This applies both to the case when the leveling system is switched on and when the leveling system is switched off. This tendency is in line with the findings in^5] and [8^, but differs from the results reported by Jia et al.^9^. However, as the error bars already indicate, the differences, e.g., for the comparison of the groups 0 rpm and 500 rpm with the leveling system switched on, are not in all cases statistically significant.

For the asphalt material tested the increase in the degree of compaction is remarkably low. For example, increasing the tamper speed from 0 rpm to 1500 rpm only leads to an increase in the degree of compaction of approx. 1.5%.

**Fig. 16:**
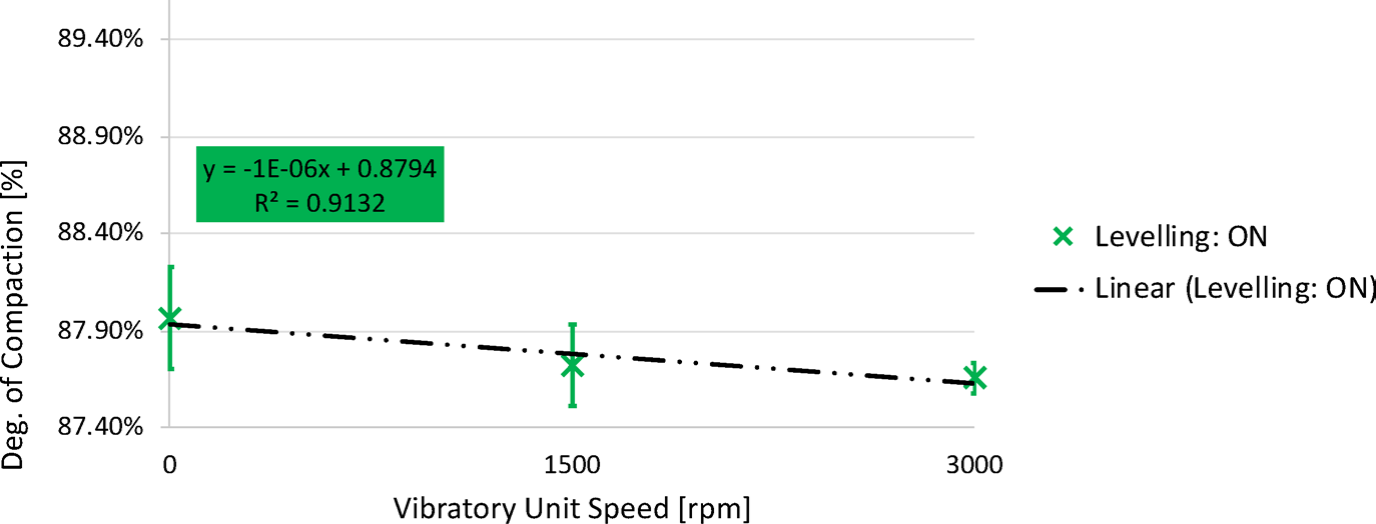
Field trial II – Degree of compaction (pre-compacted) as a function of the vibratory unit speed.

The results of the investigations into the influence of the vibratory unit on the degree of compaction can be found in Fig. 16. The mean values show a slight decrease in the degree of compaction with increasing vibratory unit speed, but the differences are not statistically significant. However, the arithmetic mean values for the respective settings do not differ statistically significantly. According to the practical understanding^11^, the vibratory unit should have little or no influence on the degree of compaction, which is consistent with the test results. However, vibratory unit results with the tamper switched off from Böhmer^5^ indicate a clear influence of the vibration unit on pre-compaction. carried out the tests (cf. Section 2.2). Wan and Jia^6^, on the other hand, also found an influence when the tamper was switched on. None of these test results agree with the results of this field tests. This could be due to many reasons, such as the use of different asphalt mixes.

### Void characteristics

**Fig. 17:**
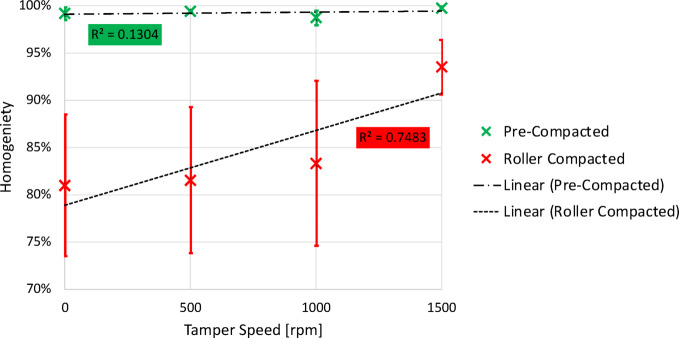
Field trial I - Homogeneity of vertical void distribution as a function of tamper speed.

To investigate the void characteristics, the homogeneity of the vertical void distribution as a function of the tamper speed is first shown in Fig. 17. Regardless of the tamper speed, a very high vertical homogeneity of at least 98.70% is achieved which aligns with the simulation based results from Harries et al.^23^. The increase in tamper speed therefore has no influence on the depth effect of the asphalt pavement in the pre-compacted state. Overall, it can be assumed that the tamper has an effect over the entire depth with the layer thicknesses installed. The homogeneity of the roller compacted side is significantly worse. The arithmetic mean is 85%. As the error bars imply, the variations between the individual samples are also significantly greater. The microsections show clear accumulations of voids, particularly in the upper and lower areas. This phenomenon was already observed in^24^ and indicates that the roller compaction is not always able to compact a very homogeneous layer produced by the screed evenly over the depth.

**Fig. 18:**
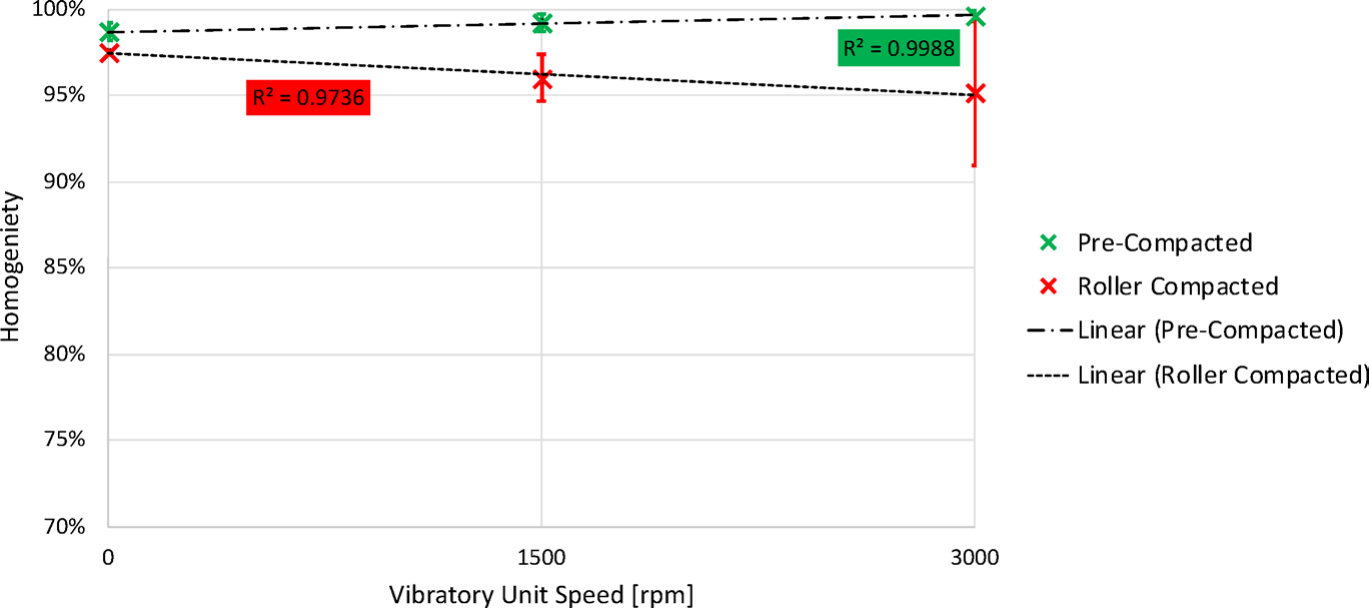
Field trial II - Homogeneity of vertical void distribution as a function of vibratory unit speed.

The results of the microstructure homogeneity as a function of the vibratory unit speed can be found in Fig. 18. Similar to the tamper, the vibratory unit speed has no verifiable influence on the vertical homogeneity. In general, a very high vertical homogeneity can be seen for the pre-compacted range. In the roller compacted area, a lower vertical homogeneity is again evident. However, this time it is much higher than in field trial I. This may be due to the different asphalt mix composition or the slightly different rolling patterns. Further investigations of the rolling effects are not the subject of this work.

**Fig. 19:**
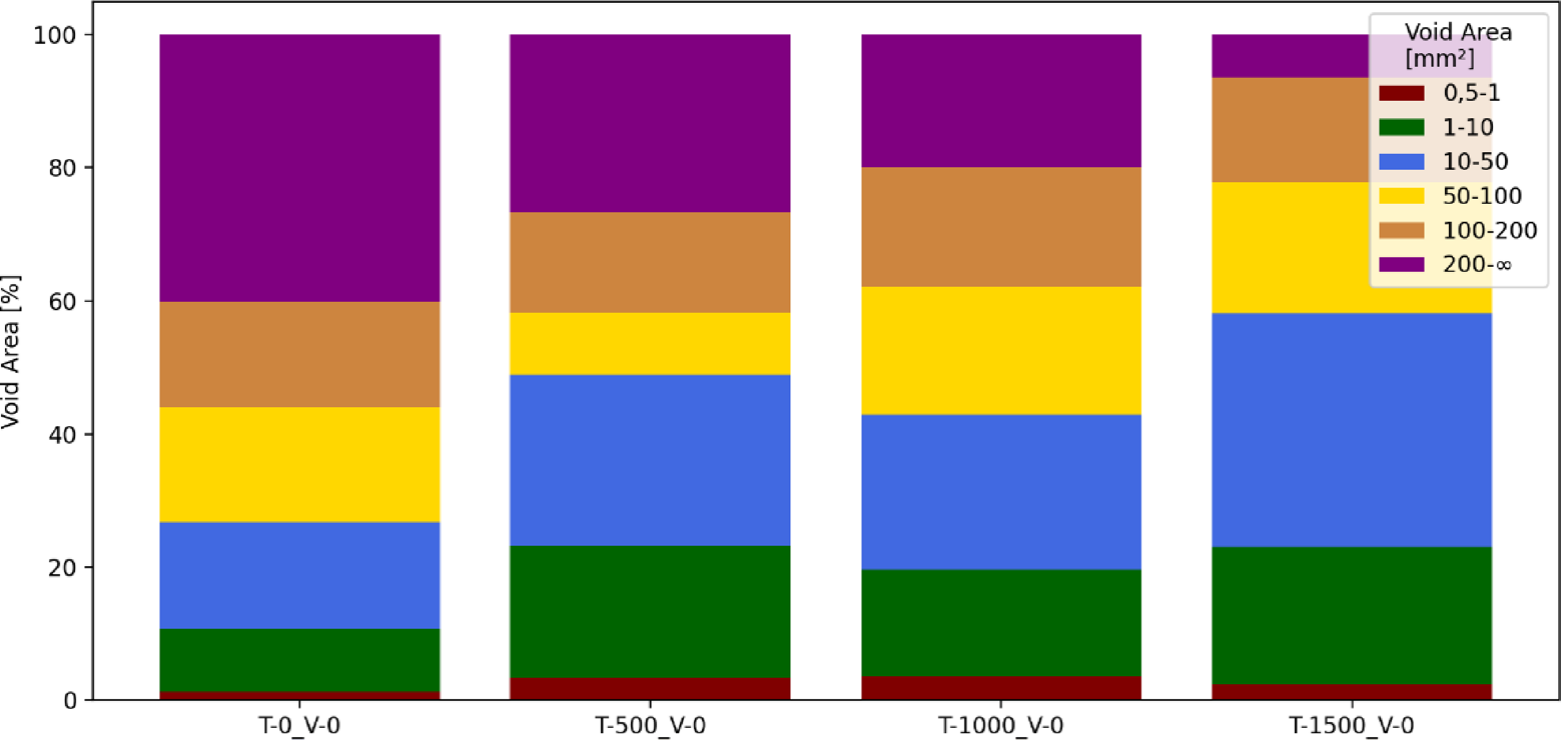
Field trial I – Pre compacted air void distribution by void classes.

**Fig. 20:**
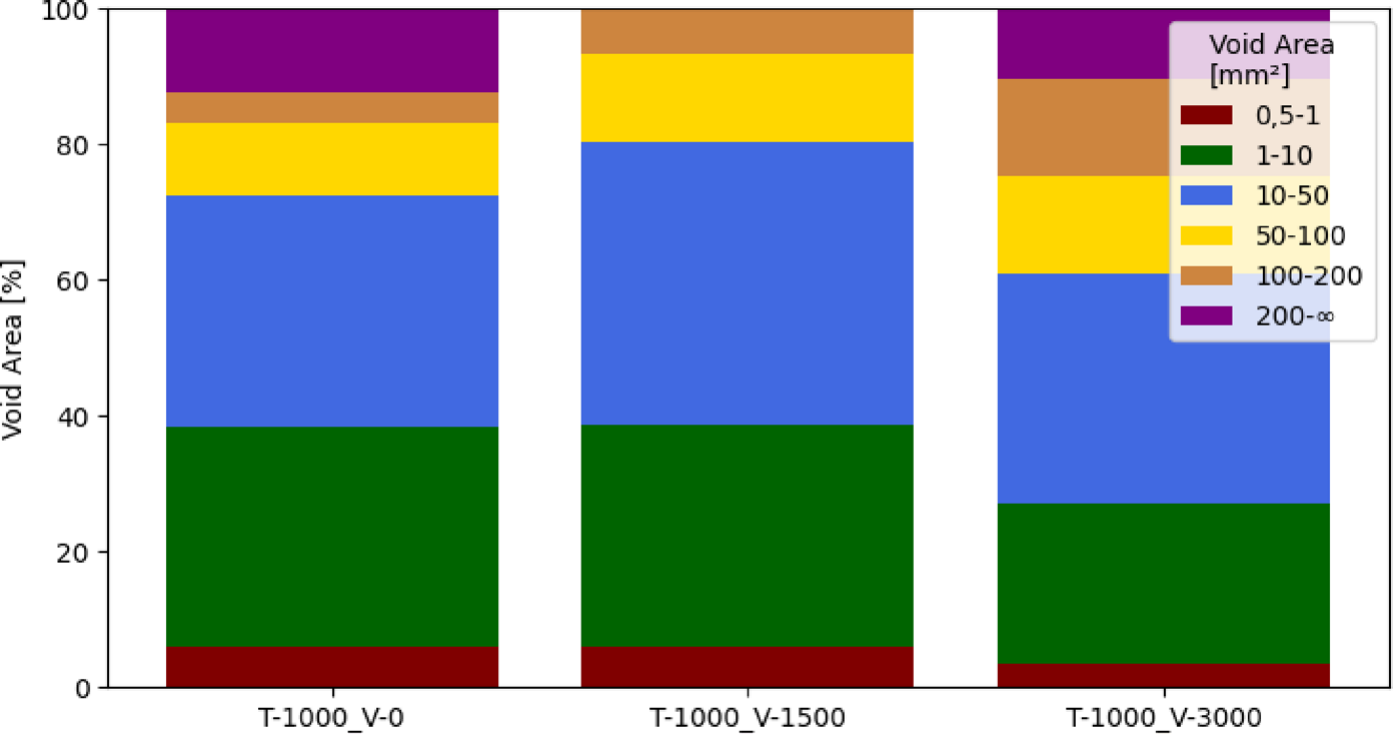
Field trial II – Pre compacted air void distribution by void classes.

Figures 19 and 20 show the influence of the tamper or vibratory unit on the cavity classes for the respective field trials. For field trial I, it can be seen that the tamper speed has a particular influence on the very large cavity areas (from 200 mm^2^). These decrease as the tamper speed increases. Smaller cavity classes increase accordingly. However, the mean values do not differ statistically significantly in most cases. The correlations should therefore be interpreted as a trend only.

The following figure shows the results for field trial II. There is no conclusive correlation between the increase in vibratory unit speed and the void area. The results did not differ statistically significantly in most cases. Based on the present data, no statistically verified effect of the vibratory unit speed on the distribution of void sizes could be identified.

**Fig. 21:**
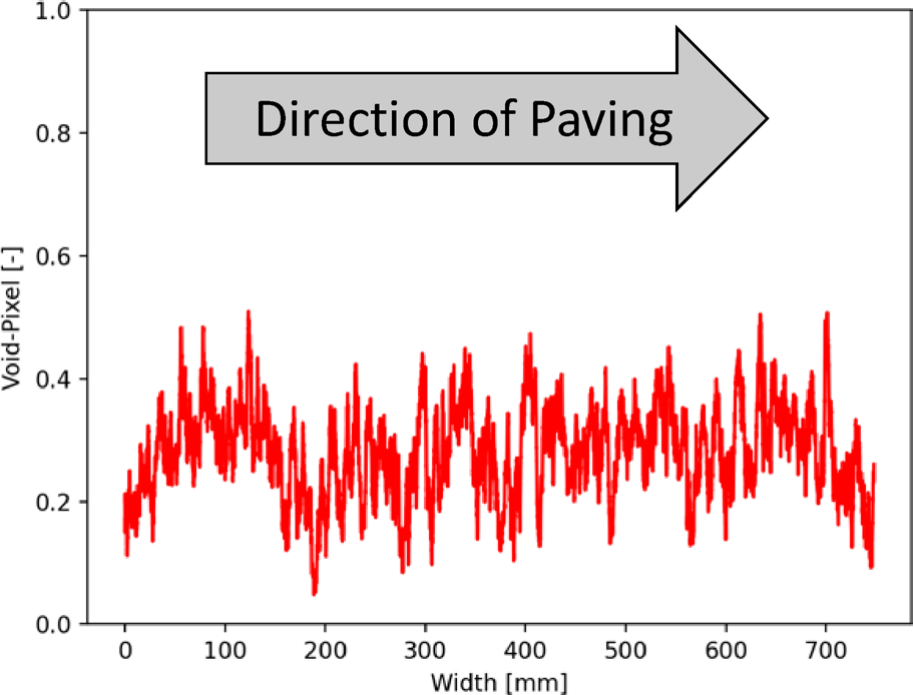
Field trial I - Horizontal cavity distribution of the removed asphalt slab at the end of the test lane L2.

Due to the problems with removing the asphalt slabs, only one of the extracted slabs could be examined with methods of asphalt petrology. Figure 21 shows the result of the horizontal void distribution. Contrary to the assumption that a decrease in the proportion of voids occurs due to the angle of attack, this could not be proven. The result may indicate that the main compaction work is carried out by the tamper and that no or only slight additional compaction is achieved under the screed plate. However, due to the limited sample size, the results shown in Fig. 21 should be interpreted as a qualitative indication only and not as a statistically verified finding.

### Surface properties

**Fig. 22:**
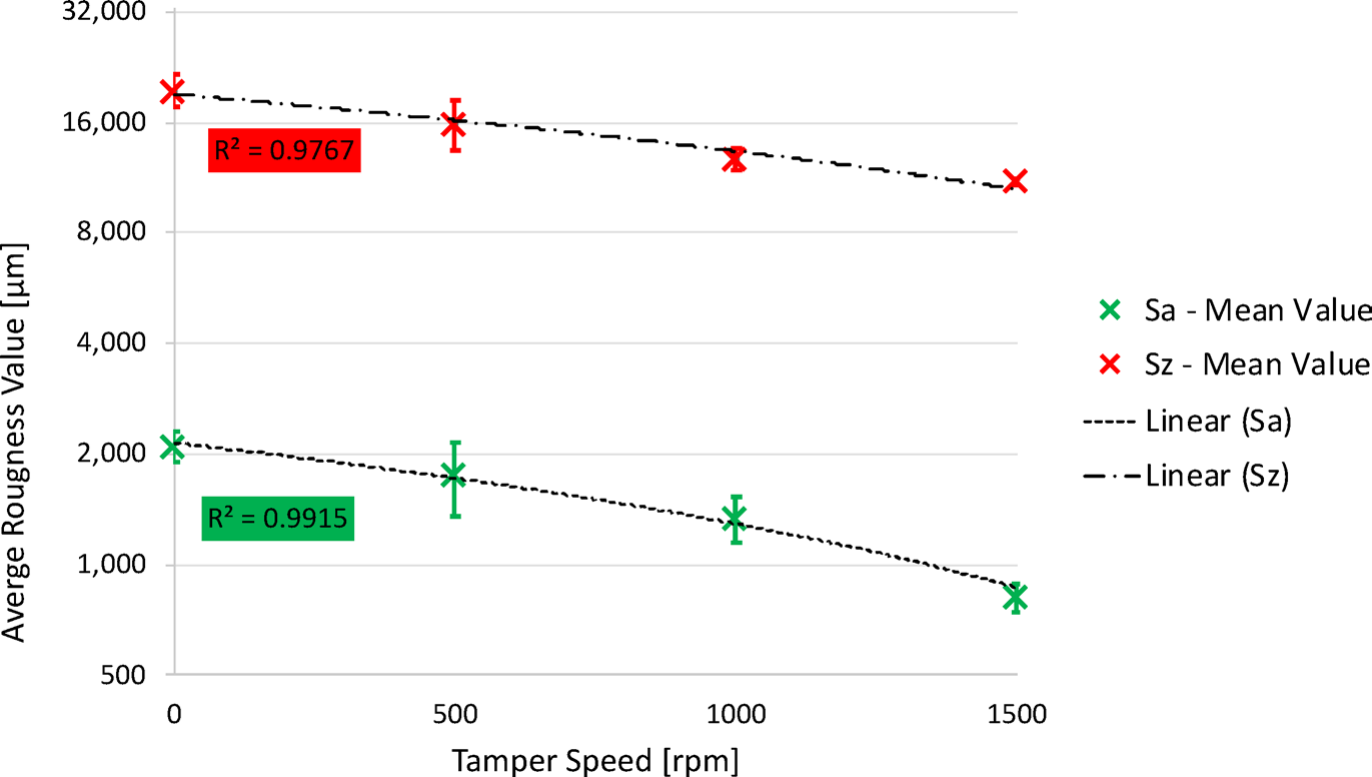
Field trial I - Roughness values as a function of the tamper speed.

The results of the surface properties can be found in Fig. 22. For both the average roughness value S_a_ and the average roughness value S_z_, the mean values indicate a decreasing roughness tendency with increasing tamper speed. This tendency is supported by high CoD, although statistically significant differences were only observed in some cases. The arithmetic mean values differ significantly in some cases. This may indicate that the grains on the surface are rearranged by the pre-compaction process in such a way that overall, less pronounced peaks and tales are created, resulting in a decrease in roughness. One explanation would be that the more often the tamper comes into contact with the asphalt material, depending on the paving speed, the more pronounced this “smoothing” effect is. However, the tamper movement itself also causes the screed plate to vibrate, which instead or additionally could promote this effect. In the literature, however, this effect is only attributed to the vibratory unit^11^.

**Fig. 23:**
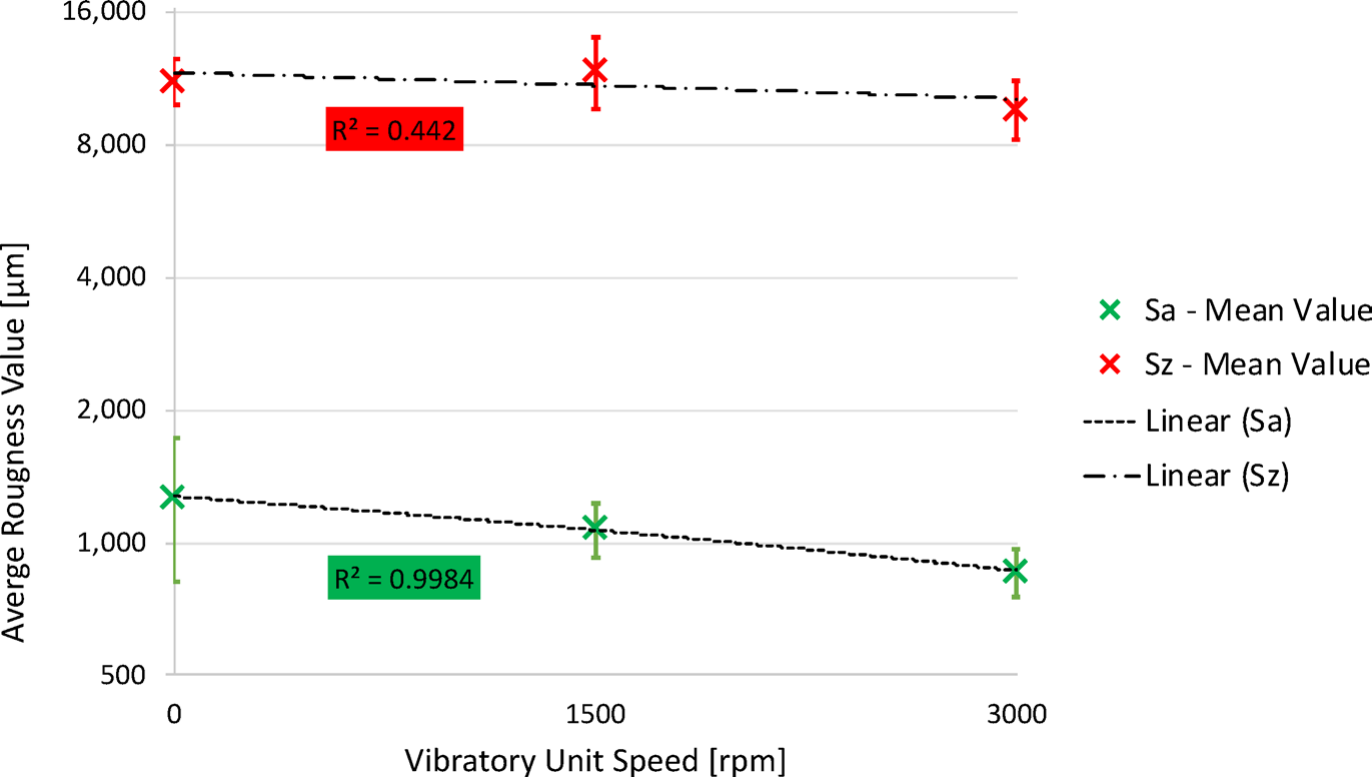
Field trial II - Roughness values as a function of the vibratory unit speed.

The results of the vibration unit tests can be found in Fig. 23. For the S_z_ value, there is only a weak correlation between roughness and vibratory unit speed. A larger coefficient of determination is found when considering at the roughness value S_a_. However, the results do not differ statistically significantly. It can therefore be concluded that no statistically significant effect of the vibratory unit speed on roughness could be identified in this field study, even though the vibratory unit speeds covered the lower and upper range of the available settings. It is reasonable to assume that the set 1000 rpm tamper speed already excites the screed to vibrate to a sufficient degree, so that further vibration excitation by the vibratory unit does not necessarily contribute to an increase in this “smoothing” effect.

## Conclusion

Two field trials were conducted with the AC 16 BN asphalt mix. The first trial examined the impact of tamper speed on the asphalt’s microstructure and surface properties. The second trial investigated the effects of vibratory unit speed. Due to paving-related deviations, particularly regarding the achieved layer thickness, the conclusions are drawn with some uncertainty. The main findings are summarized as follows:

- Increasing the tamper speed leads to a significant increase in the layer thickness (with the leveling system switched off), whereby this approaches a maximum value due to the continuously decreasing angle of attack.
- The degree of compaction k showed an increasing tendency with increasing tamper speed. No significant change in the degree of compaction could be determined for the increase in vibratory unit speed. Despite the maximum speed of the compaction units, no grain fragmentation could be detected.
- The vertical void distribution for all pre-compacted areas is very high (> 98%), so that it can be assumed that the tamper speed has no effect on the homogeneity of the paved layer. Comparative tests on roller-compacted areas have shown that the effect of the roller leads to the accumulation of voids at the top and bottom of the layer and consequently has a negative influence on the vertical homogeneity of the asphalt pavement.
- For the grain structure, hardly any statistically verified correlations could be found between the grain-to-grain distance and the influence of the tamper or vibratory unit speed. The increase in tamper speed tended to lead to a decrease in the grain-to-grain distance, which is consistent with the investigations of the degree of compaction. The evaluation of the length of contact line did not lead to any plausible results.
- By measuring the asphalt surface using a 3D camera system, it was possible to show that increasing the tamper speed tends to reduce the roughness values. No statistically significant smoothing effect could be determined for increasing vibratory unit speed at a constant tamper speed of 1000 rpm.

The measurement of the surface roughness showed that, with the tamper switched on at 1000 rpm, no significant change in surface roughness could be achieved even by substantially varying the vibratory unit speed. No positive influence on the degree of pre-compaction or the investigated microstructural parameters could be identified either. For the investigated AC 16 BN mixture and comparable paving conditions, it is therefore recommended to switch off the vibratory unit, provided that the required pre-compaction and surface quality are achieved.

Due to the clear correlation between the tamper speed and the layer thickness or the degree of pre-compaction, it is recommended to adjust the tamper speed during paving only in very exceptional cases. It is important to set the tamper as correctly as possible before the start of paving. However, due to the unknown tamper wear, the correct setting remains complex. It is therefore recommended that the wear condition of the tamper is measured before every major paving job and the setting is made accordingly.

## Data Availability

The datasets used and analyzed during the current study available from the corresponding author on reasonable request.
